# A longitudinal validation of the EQ-5D-5L and EQ-VAS stand-alone component utilising the Oxford Hip Score in the Australian hip arthroplasty population

**DOI:** 10.1186/s41687-022-00482-7

**Published:** 2022-06-20

**Authors:** D-Yin Lin, Tim Soon Cheok, Anthony J. Samson, Billingsley Kaambwa, Brigid Brown, Christopher Wilson, Hidde M. Kroon, Ruurd L. Jaarsma

**Affiliations:** 1 c/o Department of Anesthesiology, Flinders Medical Centre, Flinders Drive, Bedford Park 5042, Adelaide, SA Australia; 2 College of Medicine and Public Health, Flinders University, Adelaide, SA Australia; 3 Department of Orthopaedic and Trauma Surgery, Alice Springs Hospital, Alice Springs, NT Australia; 4 Department of Surgery, Royal Adelaide Hospital, Adelaide, SA Australia; 5 Faculty of Health and Medical Sciences, Adelaide Medical School, University of Adelaide, Adelaide, SA Australia

## Abstract

**Purpose:**

To evaluate the measurement properties of the Oxford Hip Score (OHS), EQ-5D-5L utility index and EQ-5D-5L visual analogue scale (EQ-VAS) in patients undergoing elective total hip arthroplasty in Australia.

**Methods:**

In this prospective multi-centre study, the OHS and EQ-5D-5L were collected preoperatively, six weeks (6w) and six months (6m) postoperatively. The OHS, EQ-VAS and EQ-5D-5L index were evaluated for concurrent validity, predictive validity (Spearman's Rho of predicted and observed values from a generalised linear regression model (GLM)), and responsiveness (effect size (ES) and standard response mean (SRM)).

**Results:**

362 patients were included in this analysis for 6w and 269 for 6m. The EQ-5D-5L index showed good concurrent validity with the OHS (r = 0.71 preoperatively, 0.61 at 6w and 0.59 at 6m). Predictive validity for EQ-5D-5L index was similar to OHS when regressed (GLM). Responsiveness was good at 6w (EQ-5D-5L index ES 1.53, SRM 1.40; OHS ES 2.16, SRM 1.51) and 6m (EQ-5D-5L index ES 1.88, SRM 1.70; OHS ES 3.12, SRM 2.24). The EQ-VAS returned poorer results, at 6w an ES of 0.75 (moderate) and SRM 0.8. At 6m the EQ-VAS had an ES of 0.92 and SRM of 1.00. It, however, had greater predictive validity.

**Conclusions:**

The EQ-5D-5L index and the OHS demonstrate strong concurrent validity. The EQ-5D-5L index demonstrated similar predictive validity at 6w and 6m, and both PROMs had adequate responsiveness. The EQ-VAS should be used routinely together with the EQ-5D-5L index. The EQ-5D-5L is suitable to quantify health-related quality of life in Australian hip arthroplasty patients.

**Supplementary Information:**

The online version contains supplementary material available at 10.1186/s41687-022-00482-7.

## Introduction

Total hip arthroplasty (THA) is a common operation, with 32,929 replacements performed annually in Australia (133 per 100,000) in 2017–2018 [1]. Learmonth et al. called it “the operation of the century” in *The Lancet* [2], citing improvements in quality of life following this procedure and naming cost-effectiveness as the main factor that would determine further developments in this area. Health economics and patient recovery are used as part of the evaluation of patient outcomes. Patient outcomes can be measured using patient-related outcome measures (PROMs).

The 3-level version of the EuroQol 5 Dimensions (EQ-5D-3L) is such a PROM. It is a standardized health-related quality of life (HRQoL) questionnaire that was developed in 1990 and designed to assess general health at three different levels for five dimensions [3, 4]. In 2011, it was further revised to a 5-level version (EQ-5D-5L) with five levels and five dimensions [5]. This was done to measure more nuanced differences in health response and reduce the ceiling effect. The EQ-5Dsuite of questionnaires are some of the most widely used PROMs globally. In the United Kingdom, for instance, the EQ-5D is an instrument recommended by the National Institute for Health and Clinical Excellence (NICE), the premier health technology assessment body, for calculating quality adjusted life years used in cost-utility analysis [6–8].

A validated outcome measure is one that has been tested to ensure the production of reliable, accurate results. The EQ-5D-5L has not yet been validated for the Australian orthopaedic population for HRQoL assessment. The results of the PROM are converted into ‘vectors’. These are five-digit codes representing a health state. For example, 11,111 is full health, and 55,555 represents the worst health. There are 3125 possible health states. These health states are then mapped onto a single EQ-5D-5L utility index using a country-specific value set. If a country-specific value set is not yet validated, the scores can be examined using the EQ-5D-3L value sets using a “crosswalk” method [9]. Alternatively, the generic Western Preference Pattern [10] can be used. Both of these choices come with issues related to nonspecificity and lack of validation. To date, 28 countries have validated country-specific EQ-5D-5L value sets, including England, Uruguay, Japan, Canada, The Netherlands and South Korea [11].

The EQ-VAS is a stand-alone component of the EQ-5D-5L, a rating system for the patient to self-report how they feel their general health is. The EQ-VAS is seen as a simple and unambiguous manner for a patient to communicate overall functionality and is conceptually different to the question-and-answer based nature of the rest of the PROM [12]. The Oxford Hip Score (OHS) is a PROM that was specifically developed to assess function and pain in patients undergoing a THA [13]. It has been previously utilised for assessing the concurrent validity of the EQ-5D-5L index score in THA patients in other countries [14]. A copy of the Oxford Hip Score PROM is attached as Appendix 1, while that for the EQ-5D-5L PROM is attached as Appendix 2.

This study aims to test the concurrent and predictive validity of the EQ-5D-5L (EQ-5D-5L utility index and the EQ-VAS) when compared against the OHS in the Australian hip arthroplasty population. We test concurrent and predictive validity. Concurrent validity describes the extent to which the measure to be tested correlates with an established method to measure the same. In this case, the measure to be tested is the EQ-5D-5L, and the established measurement tool is the OHS. Predictive validity describes the association between baseline and follow-up outcomes. Predictive validity is highly valued in this cohort, as this has implications for surgical suitability for individual patients. We also test responsiveness, which is defined as a measure of the sensitivity of PROMs to reflect the change in health status over time.

## Patients and methods

This multi-centre prospective study was conducted at two tertiary teaching hospitals in Australia. Orthopaedic surgeons operate routinely at both hospitals, performing approximately 500 hip arthroplasty procedures per year. Due to SARS Covid-19 related restrictions on elective operations, in 2020, this number was reduced to approximately 300 patients. The local Human Research Ethics Committee granted multi-centre approval (SALHN/329.17).

All consecutive adult patients undergoing elective total hip arthroplasty surgery were prospectively enrolled over an almost three-year period from 8th January 2018 to 1st of October 2020, with a six-month follow-up until 2nd April 2021. Informed consent was obtained from all participants. Baseline demographics were recorded for all patients, including age, gender, body mass index (BMI) and Charlson comorbidity index (CCI) [15].

Data were recorded by a dedicated research assistant, using scripted questionnaires either via telephone or via a written survey sent by postal mail. The same English language script was used at three different time points: preoperatively and six weeks and six months postoperatively. At all three time points, two validated PROMs were used: the Oxford Hip Score (OHS) [16] and the EQ-5D-5L [3] including the EQ-VAS stand-alone component. Data were entered into a password secured database and stored on the hospital computer network.

Patients were included for analysis if they had complete quality of life data. This was defined as completing the EQ-5D-5L and OHS preoperatively and at six weeks postoperatively. The validation of the EQ-5D-5L utility values was established using a discrete choice experiment approach [17].

### Oxford Hip Score

The OHS is a joint-specific PROM [18] that has been used extensively over the last 20 years [19–21]. It assesses six fields, each with 2 questions (12 questions total). These fields are pain, walking, physical activity, function, quality of life and psychological wellbeing. Each question is scored on a 5-point discrete visual analogue scale, with higher numbers correlating with better function. (Appendix 1). The final score is a total of the individual question scores. In this study, it effectively functioned as a comparative control.

### EQ-5D-5L index and EQ-VAS

The EQ-5D-5L is a standardized health-related quality of life (HRQoL) PROM that the EuroQol Group designed to quantify generic health in the adult population in the fields of mobility, self-care, usual activities, anxiety/depression and pain/discomfort. Response levels are on a 5-point scale of none, slight, moderate, severe and extreme/unable to perform. Based on Australian general population preference weights determined through a discrete choice experiment approach [17], a utility index ranging from − 0.676 to 1 can be attached to each of the EQ-5D-5L health states. Higher utilities represent better HRQoL.

The EQ-VAS is a vertical visual analogue scale that forms part of the EQ-5D-5L. It asks patients to rate their general health from 0 to 100. Higher numeric scores represent better patient function.

### Statistical analysis

All statistical analyses were performed using STATA version 17 (StataCorp, Texas, USA). Continuous variables (age, BMI, CCI) were expressed as mean and standard deviation, whereas the categorical variable (gender) was expressed as percentages (counts). A p-value of < 0.05 was considered statistically significant.

#### Concurrent validity, predictive validity and agreement

For analysis of concurrent validity, the Spearman’s correlation coefficient (rho, ρ) was utilised to compare the EQ-5D-5L index score, dimension scores of the EQ-5D-5L and EQ-VAS against the OHS. The strength of the relationship was considered low/weak (*ρ* < 0.25), fair (*ρ* = 0.25–0.50), good (*ρ* = 0.50–0.75), and excellent (*ρ* > 0.75). This magnitude of rank order correlations was sourced from previous publications on the same area [22, 23]. Predictive validity was ascertained using a regression framework whilst controlling for confounders. We utilised generalized linear models with the 6-week and 6-month postoperative PROMs as the dependant variables and preoperative values and baseline characteristics as independent variables. The average marginal effect regarding preoperative score was used to compare models if different distribution families were utilised. Agreement between the EQ-5D-5L index score and the OHS was measured using Krippendorff’s alpha, which is a reliability coefficient designed to measure the agreement among observers, coders, judges, raters, or measuring instruments [7, 24]. The following interpretations of agreement were applied: below 0.0—poor, 0.00 to 0.20—slight, 0.21 to 0.40—fair, 0.41 to 0.60—moderate, 0.61 to 0.80—substantial and 0.81 to 1.00—almost perfect [25]. Two measures of absolute agreement were considered as alternatives to Krippendorff’s alpha: Lin’s Concordance Correlation Coefficient (CCC), which is robust to departures from normality [26] and Intraclass Correlation Coefficient (ICC), with PROM data transformed using power analysis to conform to assumptions of normality and stable variance required for ICC [27–29]. The ICC was based on a two-way mixed-effect model where the individual effect was random and the effect of the instrument was fixed. Data were analyzed using Intercooled Stata software version 17.1 for Windows (Stata Corp. College Station, TX, USA). Values of the ICC and CCC higher than 0.9 were considered to indicate excellent reliability, good between 0.9 and 0.75, moderate between 0.75 and 0.5, and poor below 0.5 [27].

#### Responsiveness

Responsiveness is a measure of the sensitivity of PROMs to reflect the change in health status over time. For this study, we compared measures at baseline and at 6 weeks and 6 months follow-up using paired t-tests. Further assessment of responsiveness was quantified using effect size (ES) and standardized response mean (SRM).

Effect size was calculated using the formula:

$$ES = \frac{Mean\,Difference\,from\,Baseline}{{Standard\,Deviation\,at\,Baseline}}$$

Standard response mean was calculated using the formula:

$$SRM = \frac{Mean\,Difference\,from\,Baseline}{{Standard\,Deviation\,of\,Difference}}$$

ES and SRM were classified according to Cohen’s rule of thumb, as large (≥ 0.8), moderate (0.5–0.79) or small (< 0.5). Both ES and SRM are standardized measures of change over time in health, independent of sample size.

#### Influence of baseline characteristics on PROMs

Regression analysis using generalised linear models was performed with respect to baseline characteristics (age, gender, BMI and CCI), using the preoperative EQ-5D-5L index score, EQ-VAS and OHS as independent variables. The postoperative PROMs were used as the dependent variables. Depending on the distribution of the dependant variable, an appropriate distribution family and canonical link function were chosen. Multiple families were trialled when there was difficulty ascertaining the appropriate family of distribution, and the best fitting model was selected based on low Akaike's Information Criteria and Bayesian Information Criteria score. The coefficient, standard error and p-values were recorded.

Since the EQ-5D-5L index scores had negative values, it was determined that the Gaussian family of distribution with a canonical identity link was most appropriate. Both OHS and EQ-VAS had a non-negative distribution. Multiple families and their canonical links were fitted, including Gaussian, inverse Gaussian, Poisson, and Gamma distributions were tested for best fit. In both OHS and EQ-VAS, it was determined that the Gamma distribution provided the best fit and was hence used for the final model.

## Results

In total, 362 hip arthroplasty patients were identified from the database. These had complete data for preoperative and 6 weeks postoperatively and could be included in these two analyses. Of these, 269 were included in the study, with postoperative PROMs at 6 months available. This is due to a 26% attrition rate at 6 months.

**Fig. 1:**
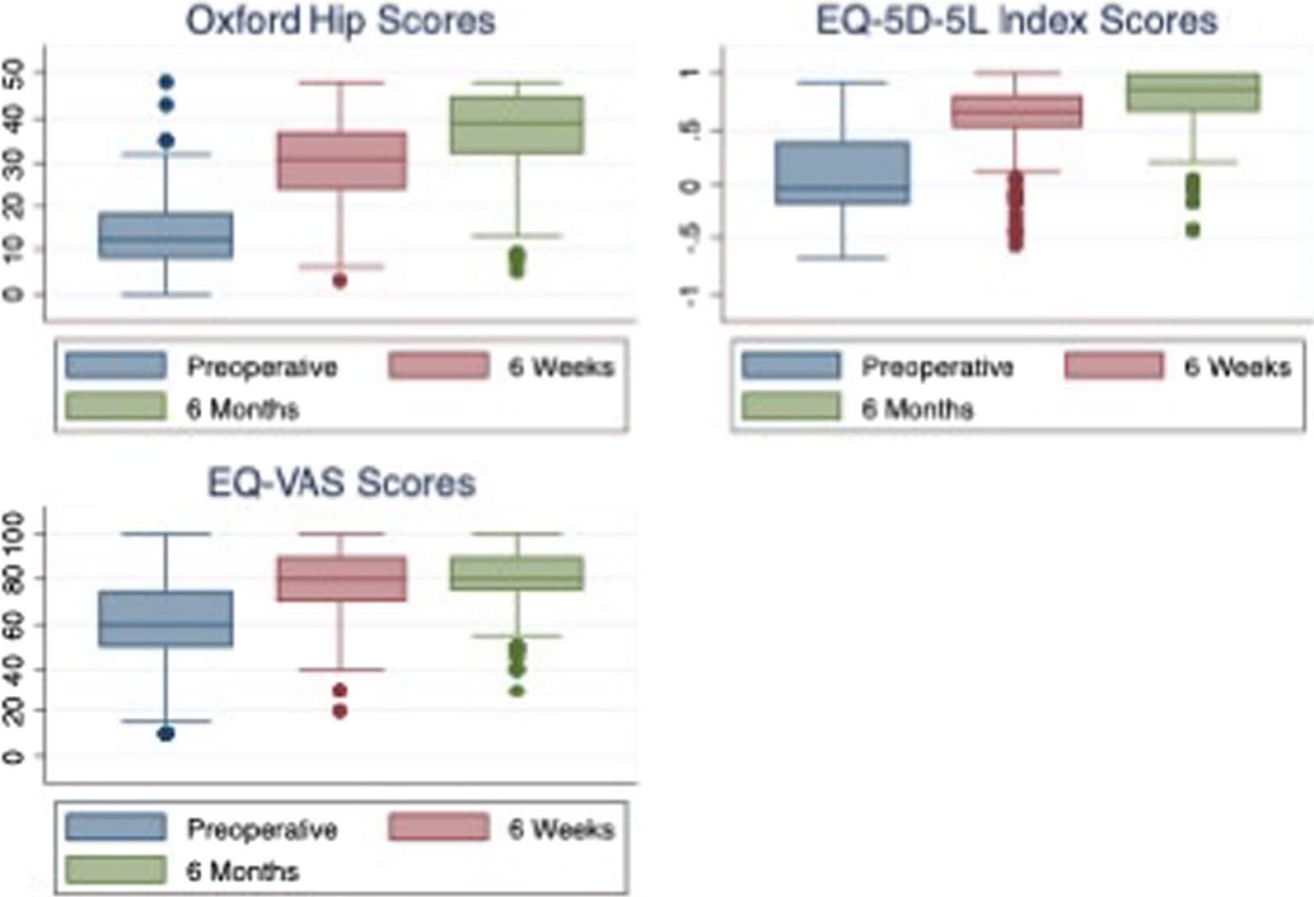
Boxplots showing distribution of PROMs scores over time

The mean age of our cohort at the time of surgery was 68.5 (SD = 12.5) years old, and 55.8% (202/362) were female. The mean preoperative BMI was 30.8 (SD = 5.6), and the mean CCI was 73.7% (SD = 22.5). A summary of baseline characteristics can be found in Table 1. Boxplots for the distributions of scores at baseline (preoperative), 6 weeks and 6 months is shown in Fig. 1.

**Table 1:** Baseline characteristics

Age (mean ± SD)	68.5 ± 12.0
Gender (M/F)	160/202
BMI (mean ± SD)	30.8 ± 5.6
CCI (mean ± SD)	73.7 ± 22.5

### Concurrent validity, predictive validity and agreement

The EQ-5D-5L index showed good concurrent validity when compared to OHS at baseline, 6 weeks, and 6 months postoperative, with a Spearman’s coefficient of 0.71, 0.61 and 0.59, respectively. EQ-VAS had good concurrent validity at 6 weeks when compared to OHS, and fair concurrent validity at baseline and 6 months, with a Spearman’s coefficient of 0.53, 0.37 and 0.45, respectively (Table 2).

**Table 2:** Concurrent and predictive validity

	EQ-5D-5L	EQ-VAS
*Concurrent validity*		
Preoperative	0.71 (good)	0.37 (fair)
6 Weeks	0.61 (good)	0.53 (good)
6 Months	0.59 (good)	0.45 (fair)

	6 Weeks		6 Months	
	Average marginal effect (standard error)	Model (link)	Average marginal effect (standard error)	Model (link)
*Predictive validity*^a^				
OHS	0.19 (0.06) (low)	Gamma (negative inverse)	0.23 (0.08) (low)	Gamma (negative inverse)
EQ-5D-5L	0.18 (0.04) (low)	Gaussian (identity)	0.16 (0.05) (low)	Gaussian (identity)
EQ-VAS	0.37 (0.04) (fair)	Gamma (negative inverse)	0.31 (0.04) (fair)	Gamma (negative inverse)

^a^Regression analysis for predictive validity was performed using generalised linear models (GLMs) with baseline characteristics age, gender, CCI and BMI as the dependent variables

*OHS* Oxford Hip Score, *EQ5D-5L* EQ-5D-5L utility index score, *EQ-VAS* visual analogue score of the EQ-5D-5L

In Table 3, the dimensions of the EQ-5D-5L index showed good concurrent validity when compared to the corresponding OHS at baseline, 6 weeks, and 6 months postoperative, with a Spearman’s coefficient ranging from 0.52 to 0.62 (good) for Mobility, Self-Care, Usual Activities and Pain. Concurrent validity was only fair for the Anxiety/Depression dimension, with a Spearman’s coefficient of 0.28 (preoperative), 0.33 (6 weeks) and 0.37 (6 months).

**Table 3:** OHS as compared to EQ-5D-5L dimensional components over time (Spearman’s correlation)

	EQ-5D-5L dimensions				
	Mobility	Self-care	Usual activities	Pain /discomfort	Anxiety/depression
OHS—preoperative	0.6171 (good)	0.5302 (good)	0.6342 (good)	0.6677 (good)	0.2822 (fair)
OHS—6 weeks	0.5639 (good)	0.5065 (good)	0.5934 (good)	0.5881 (good)	0.3328 (fair)
OHS—6 months	0.5564 (good)	0.5296 (good)	0.5725 (good)	0.5182 (good)	0.3673 (fair)

*OHS* Oxford Hip Score, *EQ5D-5L* EuroQol 5 dimensions 5 level instrument

The predictive validity of each score generated by the three different scores was determined using generalized linear models, with regression to baseline scores and covariates. In all cases, the distribution that provided the best model fit was the Gamma distribution with a canonical negative inverse link. The average marginal effects for the preoperative score were recorded and displayed in Table 2. The EQ-5D-5L index score showed similar predictive validity when compared to OHS at 6 weeks (average marginal effect of 0.19 and 0.18 respectively) and 6 months (average marginal effect of 0.23 and 0.16 respectively). However, EQ-VAS showed greater predictive validity than both OHS and EQ-5D-5L index score at 6 weeks and 6 months (average marginal effect of 0.37 and 0.31 respectively).

As shown in Table 4, the agreement between the EQ-5D-5L utility and OHS total scores ranged from moderate to substantial/good when measured using all three agreement indices (Krippendorff’s alpha, ICC and CCC). The best agreement was seen at the preoperative stage, while the least agreement was at 6 weeks. There was less agreement between the EQ-VAS and OHS total scores, ranging from poor/fair to moderate. The best agreement was seen at 6 weeks, while the least agreement was at the preoperative stage.

**Table 4:** Measuring agreement between the PROMs

		EQ-5D-5L vs OHS	EQ-VAS vs OHS
Preoperative	Krippendorff’s alpha	0.704 (0.648, 0.752)—substantial	0.382 (0.289, 0.465)—fair
	ICC	0.827 (0.787, 0.859)—good	0.553 (0.450, 0.636)—moderate
	CCC	0.704 (0.652, 0.756)—moderate	0.381 (0.292, 0.469)—poor
6 weeks	Krippendorff’s alpha	0.640 (0.575, 0.697)—substantial	0.519 (0.439, 0.590)—moderate
	ICC	0.781 (0.731, 0.822)—good	0.684 (0.611, 0.743)—moderate
	CCC	0.640 (0.579, 0.701)—moderate	0.519 (0.443, 0.594)—moderate
6 months	Krippendorff’s alpha	0.658 (0.583, 0.719)—substantial	0.462 (0.361, 0.550)—acceptable
	ICC	0.793 (0.737, 0.837)—good	0.632 (0.532, 0.710)—moderate
	CCC	0.656 (0.588, 0.725)—moderate	0.461 (0.366, 0.555)—poor

*PROMs* patient related outcome measures, *OHS* Oxford Hip Score, *EQ5D-5L* EQ-5D-5L utility index score, *EQ-VAS* Visual analogue score of the EQ-5D-5L, *ICC* intraclass correlation coefficient, *CCC* Lin’s concordance correlation coefficient

### Responsiveness

These findings are detailed in Table 5. At 6 weeks, all three PROMs showed significant differences between baseline and follow-up scores. Both OHS and EQ-5D-5L had a large ES and SRM. The ES for OHS and EQ-5D-5L index was 2.16 and 1.53, respectively, and the SRM was 1.51 and 1.40, respectively, p < 0.0001. The EQ-VAS had a moderate ES of 0.75 and a large SRM of 0.80, p < 0.0001.

**Table 5:** Responsiveness of PROMs

	Preoperative	6 Weeks	Mean difference	Paired t-test	Effect size	Standard response mean
*(a) 6 weeks*						
OHS	13.73 ± 7.32	30.10 ± 9.08	16.37 ± 10.85	< 0.0001	2.16 (large)	1.51 (large)
EQ-5D-5L	0.08 ± 0.36	0.63 ± 0.27	0.56 ± 0.40	< 0.0001	1.53 (large)	1.40 (large)
EQ-VAS	61.57 ± 20.11	76.64 ± 15.83	15.07 ± 18.82	< 0.0001	0.75 (moderate)	0.80 (large)

	Preoperative	6 Months	Mean difference	Paired t-test	Effect size	Standard response mean
*(b) 6 Months*						
OHS	13.71 ± 7.59	37.41 ± 9.23	23.70 ± 10.56	< 0.0001	3.12 (large)	2.24 (large)
EQ-5D-5L	0.10 ± 0.37	0.78 ± 0.26	0.69 ± 0.40	< 0.0001	1.88 (large)	1.70 (large)
EQ-VAS	62.00 ± 20.19	80.59 ± 13.90	18.59 ± 18.64	< 0.0001	0.92 (large)	1.00 (large)

*PROMs* patient reported outcome measures, *OHS* Oxford Hip Score, *EQ5D-5L* EQ-5D-5L utility index score, *EQ-VAS* Visual analogue score of the EQ-5D-5L

At 6 months, all three PROMs again showed a significant difference between baseline and follow-up scores: The ES for OHS, EQ-5D-5L index and EQ-VAS was 3.12, 1.88 and 0.92, respectively, and the SRM was 2.24, 1.70 and 1.00, respectively.

### Influence of baseline characteristics on PROMs

There was a statistically significant positive association between higher preoperative OHS scores on one end and both male gender and BMI. However, higher EQ-5D-5L index and EQ-VAS scores were only significantly associated with higher BMI and male gender, respectively.

## Discussion

This analysis is an empirical validation of the EQ-5D-5L for suitability of HRQoL assessment for hip arthroplasty patients using experienced-based patient data from a prospective multi-centre study database, with the correlation between the Oxford Hip Scores, EQ-VAS, and the EQ-5D-5L PROMs examined. The findings support the utilization of EQ-5D-5L index score as a valid and reliable instrument in assessing HRQoL amongst these patients.

The limits of agreement were good between the EQ-5D-5L index score and the OHS, and they can be considered similar to each other in terms of concurrent validity. However, the OHS is a joint-specific PROM, whereas the EQ-5D-5L index score is designed to assess overall functionality. For example, someone who can compensate enough to perform daily tasks and cope well with the mental burden of an arthritic hip on the EQ-5D-5L index score, may record gait disturbances and set specific difficulties with mobility on the OHS. We chose the OHS as a comparator for this validation as it is a widely used PROM, with significant overlap in terms of items with the EQ-5D-5L index score. For example, both feature mobility, pain/discomfort and usual activities. This was shown in more detail when the OHS was compared against the dimensions of the EQ-5D-5L. There is a high degree of correlation between the dimensions for the EQ-5D-5L and the OHS for the most part. The exception is the relationship between the Anxiety/Depression dimension of the EQ-5D-5L and the OHS, where the correlation is only fair. This is in line with evidence from the literature [30–32] that shows that strong correlations exist between instruments and dimensions that measured similar constructs. Hence, they should be utilised concurrently to complement each other, instead of being considered as substitutes for one another.

The longitudinal nature of this study with multiple time points lends itself well to assessing incremental changes in the population and detecting differences in the performance of both PROMs. The experience-based and prospective nature of this data is also a strong point.

The EQ-VAS as a stand-alone measure showed a smaller ES than the EQ-5D-5L index score at both six weeks (0.75 versus 1.53 respectively, p < 0.0001) and six months (0.80 versus 1.40 respectively, p < 0.0001). SRM was also large for both scores at the six-week and six-month time points. However, the EQ-VAS has better predictive validity than the EQ-5D-5L index score and OHS. This suggests that it has a higher predictive value for postoperative recovery and should be routinely used as an adjunct to the EQ-5D-5L index score. A reason for this better predictive validity may be the much broader nature of the VAS (i.e. not proscribed by the domains or items as in the OHS or EQ-5D-5L descriptive system) which allows the patients to include more in their subjective rating of health. This is beneficial for patient stratification and counselling with regards to realistic rehabilitation expectations and postsurgical results.

An assessment of the agreement between the EQ-5D-5L and the EQ-VAS on one hand and the OHS on the other showed acceptable agreement (moderate to good/substantial for most comparisons). This suggests that while the ratings from the instruments were not identical, they were moderately close and should be considered complements rather than substitutes of one another.

Some limitations of this study have to be addressed. There were approximately 25% missing data for patients at six months. Therefore, these patients had to be excluded, introducing a response bias. There was also an incomplete recording of patients’ baseline characteristics, with 90.6% (328/362) patients having their BMI recorded and 94.2% (341/362) having their CCI recorded.

## Conclusions

In conclusion, The EQ-5D-5L index score and the Oxford Hip Score demonstrate good concurrent validity in this study. The EQ-5D-5L index score revealed a large effect size at six weeks and six months postoperatively, and both PROMs had adequate responsiveness. The EQ-5D-5L index score PROM is suitable to quantify general health-related quality of life in the Australian hip arthroplasty patient population.

## Supplementary Information

**Additional file 1.** Oxford Hip Score.

**Additional file 2.** EQ-5D-5L.

## Data Availability

Data available upon request.

## Abbreviations

THA: Total hip arthroplasty
6w: Six weeks
6m: Six months
ES: Effect size
SRM: Standardized response mean
PROMs: Patient reported outcome measures
OHS: Oxford Hip Score
VAS: Visual analogue scale
CCI: Charleson Comorbidity Index
HRQoL: Health Related Quality of Life
TMLE: Targeted maximum likelihood estimation
BMI: Body mass index
TTO: Time trade off
