# Supplementary material for: A longitudinal validation of the EQ-5D-5L and EQ-VAS stand-alone component utilising the Oxford Hip Score in the Australian hip arthroplasty population
Source: J Patient Rep Outcomes. 2022 Jun 20;6:71. doi: 10.1186/s41687-022-00482-7 (PMC9207851; doi:10.1186/s41687-022-00482-7)
Supplement: Supplementary file 1 — Additional file 1. Oxford Hip Score. [file 41687_2022_482_MOESM1_ESM.pdf]

Problems with your hip

During the past 4 weeks..

✓**tick <u>one</u> box for <u>every</u> question.**

1. ***During the past 4 weeks***........

How would you describe the pain you usually had from your hip?

None	Very mild	Mild	Moderate	Severe
❑	❑	❑	❑	❑

2. ***During the past 4 weeks***........

Have you had any trouble with washing and drying yourself (all over) because of your hip?

No trouble at all	Very little trouble	Moderate trouble	Extreme difficulty	Impossible to do
❑	❑	❑	❑	❑

3. ***During the past 4 weeks***........

Have you had any trouble getting in and out of a car or using public transport because of your hip? *(whichever you tend to use)*

No trouble at all	Very little trouble	Moderate trouble	Extreme difficulty	Impossible to do
❑	❑	❑	❑	❑

4. ***During the past 4 weeks***........

Have you been able to put on a pair of socks, stockings or tights?

Yes, Easily	With little difficulty	With moderate difficulty	With extreme difficulty	No, Impossible
❑	❑	❑	❑	❑

5. ***During the past 4 weeks***........

Could you do the household shopping on your own?

Yes, Easily	With little difficulty	With moderate difficulty	With extreme difficulty	No, Impossible
❑	❑	❑	❑	❑

6. ***During the past 4 weeks***........

For how long have you been able to walk before pain from your hip becomes severe? (*with or without a stick)*

No pain/ More than 30 minutes	16 to 30 minutes	5 to 15 minutes	Around the house only	Not at all -pain severe on walking
❑	❑	❑	❑	❑

The Oxford Hip Score©Department of Public Health, University of Oxford, Old Road Campus, Oxford OX3 7LF , UK.

P.T.O./

During the past 4 weeks...

✓**tick <u>one</u> box for <u>every</u> question**

**7**	***During the past 4 weeks**........*<br>Have you been able to climb a flight of stairs?<br>Yes, Easily ❑ · With little difficulty ❑ · With moderate difficulty ❑ · With extreme difficulty ❑ · No, Impossible ❑
**8**	***During the past 4 weeks**........*<br>After a meal (sat at a table), how painful has it been for you to stand up from a chair <u>because of your hip</u>?<br>Not at all painful ❑ · Slightly painful ❑ · Moderately painful ❑ · Very painful ❑ · Unbearable ❑
**9**	***During the past 4 weeks**......*<br>Have you been limping when walking, <u>because of your hip</u>?<br>Rarely/ never ❑ · Sometimes, or just at first ❑ · Often, not just at first ❑ · Most of the time ❑ · All of the time ❑
**10**	***During the past 4 weeks**........*<br>Have you had any sudden, <u>severe</u> pain - 'shooting', 'stabbing' or 'spasms' - <u>from the affected hip</u>?<br>No days ❑ · Only 1 or 2 days ❑ · Some days ❑ · Most days ❑ · Every day ❑
**11**	***During the past 4 weeks**........*<br>How much has <u>pain from your hip</u> interfered with your usual work *(including housework)*?<br>Not at all ❑ · A little bit ❑ · Moderately ❑ · Greatly ❑ · Totally ❑
**12**	***During the past 4 weeks**........*<br>Have you been troubled by <u>pain from your hip</u> in bed at night?<br>No nights ❑ · Only 1 or 2 nights ❑ · Some nights ❑ · Most nights ❑ · Every night ❑

©Department of Public Health, University of Oxford, Old Road Campus, Oxford OX3 7LF , UK.
