# Supplementary material for: A longitudinal validation of the EQ-5D-5L and EQ-VAS stand-alone component utilising the Oxford Hip Score in the Australian hip arthroplasty population
Source: J Patient Rep Outcomes. 2022 Jun 20;6:71. doi: 10.1186/s41687-022-00482-7 (PMC9207851; doi:10.1186/s41687-022-00482-7)
Supplement: Supplementary file 2 — Additional file 2. EQ-5D-5L. [file 41687_2022_482_MOESM2_ESM.pdf]

Health Questionnaire (EQ-5D-5L)

Under each heading, please tick the ONE box that best describes your health TODAY.

MOBILITY

☐ 1 I have no problems in walking about
☐ 2 I have slight problems in walking about
☐ 3 I have moderate problems in walking about
☐ 4 I have severe problems in walking about
☐ 5 I am unable to walk about

SELF-CARE

☐ 1 I have no problems washing or dressing myself
☐ 2 I have slight problems washing or dressing myself
☐ 3 I have moderate problems washing or dressing myself
☐ 4 I have severe problems washing or dressing myself
☐ 5 I am unable to wash or dress myself

USUAL ACTIVITIES *(e.g. work, study, housework, family or leisure activities)*

☐ 1 I have no problems doing my usual activities
☐ 2 I have slight problems doing my usual activities
☐ 3 I have moderate problems doing my usual activities
☐ 4 I have severe problems doing my usual activities
☐ 5 I am unable to do my usual activities

PAIN / DISCOMFORT

☐ 1 I have no pain or discomfort
☐ 2 I have slight pain or discomfort
☐ 3 I have moderate pain or discomfort
☐ 4 I have severe pain or discomfort
☐ 5 I have extreme pain or discomfort

ANXIETY / DEPRESSION

☐ 1 I am not anxious or depressed
☐ 2 I am slightly anxious or depressed
☐ 3 I am moderately anxious or depressed
☐ 4 I am severely anxious or depressed
☐ 5 I am extremely anxious or depressed

UK (English) © 2009 EuroQol Group EQ-5D™ is a trade mark of the EuroQol Group

Health Questionnaire (EQ-5D-5L)

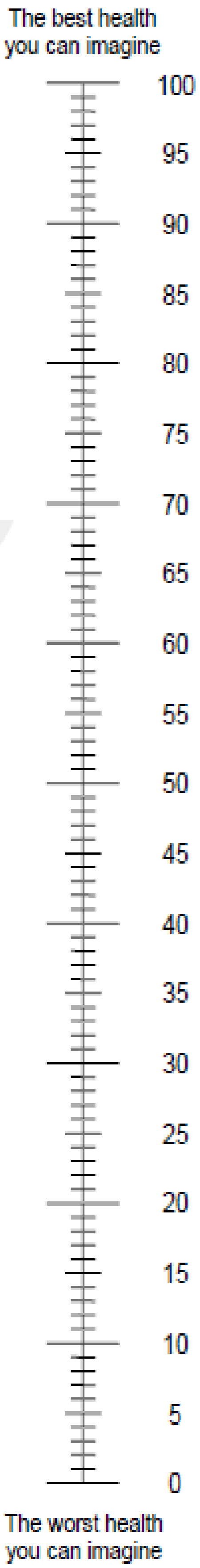

- We would like to know how good or bad your health is **TODAY**.
- This scale is numbered from 0 to 100.
- 100 means the <u>best</u> health you can imagine.
  0 means the <u>worst</u> health you can imagine.
- Mark an X on the scale to indicate how your health is **TODAY**
- Now, please write the number you marked on the scale in the

  below.

YOUR HEALTH TODAY = [ ]

UK (English) © 2009 EuroQol Group EQ-5D™ is a trade mark of the EuroQol Group
